# Aflatoxins in Wheat Grains: Detection and Detoxification through Chemical, Physical, and Biological Means

**DOI:** 10.3390/life14040535

**Published:** 2024-04-22

**Authors:** Ahmed Mahmoud Ismail, Muhammad Hassan Raza, Naseem Zahra, Rafiq Ahmad, Yasar Sajjad, Sabaz Ali Khan

**Affiliations:** 1 Department of Arid Land Agriculture, College of Agricultural and Food Sciences, King Faisal University, P.O. Box 420, Al-Ahsa 31982, Saudi Arabia; 2 Pests and Plant Diseases Unit, College of Agricultural and Food Sciences, King Faisal University, P.O. Box 420, Al-Ahsa 31982, Saudi Arabia; 3 Vegetable Diseases Research Department, Plant Pathology Research Institute, Agricultural Research Center (ARC), Giza 12619, Egypt; 4 Department of Biotechnology, COMSATS University Islamabad-Abbottabad Campus, Abbottabad 22060, Pakistan; hassanraza4799@gmail.com (M.H.R.); drrafiq@cuiatd.edu.pk (R.A.); yasarsajjad@cuiatd.edu.pk (Y.S.); 5 Food and Biotechnology Research Centre, PCSIR Laboratories Complex, Ferozepur Road, Lahore 54600, Pakistan; drnaseemzahra@gmail.com

**Keywords:** *Aspergillus*, biological, ELISA, mycotoxins, physicochemical, *Triticum aestivum*

## Abstract

Wheat (*Triticum aestivum* L.) is an essential food crop in terms of consumption as well as production. Aflatoxin exposure has a widespread public health impact in economically developing nations, so there is a need to establish preventive techniques for these high-risk populations. Pre-harvest and post-harvest practices are the two strategies used to control aflatoxin contamination, which include the use of genetically modified crops that show resistance against *Aspergillus* infection, the use of pesticides, changing the planting and harvesting time of crops, and physical, chemical, and biological methods. In this research, aflatoxin detection and quantification were performed in different wheat varieties to determine quantitative differences in comparison to the European Commission’s limit of 4 ppb aflatoxins in wheat. TLC for qualitative and the ELISA kit method for quantitative analysis of aflatoxins were used. Out of 56 samples, 35 were found contaminated with aflatoxins, while the remaining 21 samples did not show any presence of aflatoxins. Out of the 35 contaminated samples, 20 samples showed aflatoxin contamination within the permissible limit, while the remaining 15 samples showed aflatoxin concentration beyond the permissible level, ranging from 0.49 to 20.56 ppb. After quantification, the nine highly contaminated wheat samples were detoxified using physical, chemical, and biological methods. The efficiency of these methods was assessed, and they showed a significant reduction in aflatoxins of 53–72%, 79–88%, and 80–88%, respectively. In conclusion, the difference in aflatoxin concentration in different wheat varieties could be due to genetic variations. Furthermore, biological treatment could be the method of choice for detoxification of aflatoxins in wheat as it greatly reduced the aflatoxin concentration with no harmful effect on the quality of the grains.

## 1. Introduction

Wheat is one of the most important staple crops grown globally, including in Pakistan. Wheat belongs to the Poaceae family, which is the most diverse family of flowering plants with over 10,000 species [1]. The genus *Triticum* includes various types of wheat, with common wheat (*Triticum aestivum* L.), having a hexaploid genome (2n = 6x = 42), being the most extensively cultivated one [2,3]. The genome size of wheat is 17 billion base pairs with 124,000 genes.

Based on the growing season, wheat can be classified into winter and spring types. From 1990 to 2021, the amount of wheat produced worldwide increased from 592 to 788.6 million metric tons [4]. The top-producing nations of wheat are Pakistan, Ukraine, China, the European Union, Argentina, Russia, the United States, India, Canada, and Türkiye. Pakistan produces about 3.04% of global wheat production from an area of 3.57% of the world’s, and is ranked seventh in the world [5]. Due to its most abundant agricultural land, Punjab produces around 76% of the country’s wheat, Khyber Pakhtunkhwa produces 5%, Sindh contributes 16%, and about 3% comes from Balochistan [6]. Although it can grow at temperatures of 4–35 °C, wheat germinates best at around 12–25 °C.

Aflatoxins are a group of hazardous metabolites produced by fungi (*A. flavus* and *A. parasiticus*) that are present in nature at times of drought, heat, and humidity [7]. Aflatoxins were first detected in the UK in 1961 after *Aspergillus flavus*-contaminated feed caused the deaths of more than a million turkeys [8].

Aflatoxins B1, B2, G1, and G2 are the four main types of aflatoxins. AFM1 is a variant of AFB1 and is primarily found in milk and other dairy products. The ideal temperature for the growth of aflatoxins is 33 °C, and they grow in warm and humid climates [9]. Aflatoxin exposure can result in malignancies of the liver and other organs. Cereals, ground nuts, oil seeds, and grains are the main sources of aflatoxin growth [10].

About 25% of people who die from acute aflatoxicosis have had a significant exposure to aflatoxin [11]. Children are particularly susceptible to aflatoxin exposure since their immune systems are less developed than those of adults [12]. Foods and feeds containing aflatoxin pose a risk to both human and animal health. Aflatoxins have been proven to have a variety of negative consequences on health, including liver toxicity, mutagenesis, tumorigenesis, immunosuppression, effects on the epigenome, reproductive issues, and growth retardation [13]. Aflatoxin appears to interact with DNA, causing damage, which is dangerous because if the DNA is not repaired, a mutation may arise, which may be the first step in the series of events leading to cancer. As a result of the findings of numerous investigations, the International Agency for Research on Cancer (IARC) has categorized aflatoxin B1 as a Group 1 human carcinogen [14].

Around the world, poor storage techniques and a lack of technical capacity have led to 50–60% losses of cereal grains. Every year, Pakistan loses USD 76 to 90 million due to insufficient facilities for wheat grain storage [15]. Thailand, Indonesia, and the Philippines combined have been estimated to lose USD 1 billion each year due to the presence of different mycotoxins. There are different analytical techniques that are being used for the analysis of aflatoxins such as TLC, HPLC, GC, ELISA, and LC-MS [16]. Due to high presence of aflatoxins in food and feed, a variety of strategies have been developed to remove contamination and to restore food quality and edibility. Pre-harvest and post-harvest are the two types of control strategies [17]. Pre-harvest strategies include the use of genetically modified crops that show resistance against *Aspergillus* infection, the use of pesticides, and changing the planting time of crops [18]. Post-harvest strategies include physical, chemical, and biological methods. In this research, an attempt has been made to quantify the aflatoxins in various wheat varieties grown in Pakistan to determine whether they exceed the permissible limit set by the European Community’s Scientific Commission and to determine which method is better for the control of aflatoxins in wheat grains.

## 2. Materials and Methods

### 2.1. Sample Collection

Seeds of 16 different wheat varieties (Dilkash, Shahkar-2013, Pirsabak-2015, Zincol-2016, Paseena-2017, Kohat-2017, Khaista-2017, Gulzar-2019, Markaz-2019, Fahim-2019, Pirsabak-2019, Pirsabak-2021, Subhani-2021, Abaseen-2021, Zarghan-2021, Taskeen-2022) were obtained from Barani Agricultural Research Station Kohat and were sown in the field area of COMSATS University Islamabad, Abbottabad Campus. Wheat samples (1 kg of seeds) of each variety were then collected. Also, 40 random samples from District Sahiwal, Punjab were collected. These samples were placed in airtight plastic bags and transferred to FBRC, PCSIR Laboratories Complex, Lahore for quantitative and qualitative determination and detoxification of aflatoxins.

### 2.2. Detection of Aflatoxins by Thin-Layer Chromatography

#### 2.2.1. Sample Preparation

Accurately weighed samples of fifty grams of wheat seeds were ground to make a fine powder. This powder was put in a conical flask containing 250 mL chloroform and 25 mL distilled water. These flasks were then put on a wrist-action shaker for 30 min [19]. The sample extracts were then filtered in beakers using Whatman filter paper no. 4, and 50 mL of each sample extract was collected in beakers. These beakers were put on a hot plate at 250 °C and were dried [20]. All the reagents were bought from a local vendor of Province Punjab, Pakistan.

#### 2.2.2. Spotting and Visualization

The spotting dilutions were obtained in microliters. With a microsyringe, a 25 µL spot of the test solution was put on the thin-layer chromatography plate. A standard spot of 5 or 10 µL of aflatoxins was applied on the same plate as an internal standard. Anhydrous ether was used to partially develop the plate in the thin-layer chromatographic tank before it was removed and dried. Then, a thin-layer chromatographic tank with acetone-chloroform (1:9) was used to develop the plate again in the same position [21]. After that, the plate was air-dried, and it was visualized under UV light for the presence or absence of aflatoxins. The fluorescence of sample spots was compared to that of aflatoxin spots.

### 2.3. Detection and Quantification of Aflatoxins by ELISA

#### 2.3.1. Sample Preparation

To prepare samples for ELISA, 5 g of wheat seeds was ground and added to conical flasks containing 25 mL of 70% (*v*/*v*) methanol. These flasks were vigorously shaken for 30 min, and the extract was filtered out (5 mL) using Whatman filter paper no. 4 [22].

#### 2.3.2. Pipetting and Visualization

The quantifiable analysis of aflatoxins in the samples was performed using Neogen Veratox^®^ Aflatoxin Test Kit, Product 8030, Neogen, Lansing, MI, USA. Before doing so, all the reagents were warmed at room temperature. One mixing microwell was removed for each sample to be tested, and four microwells for controls were placed in the microwell holder. The strip was placed in the well holder after marking one end. Prior to use, each reagent was swirled to mix it. A total of 100 µL of conjugate was added to red-marked mixing wells. Then, 100 µL of sample was added to the same mixing wells. The controls were added to separate wells. After that, the liquid in the wells was mixed by pipetting it up and down three times. Then, 100 µL of this liquid was transferred to the antibody-coated wells and incubated at room temperature for two minutes. The wells were filled with distilled water, which was then removed. This step was repeated five times, after which the wells were dried with a paper towel to remove any remaining water. Then, 100 µL of substrate was added to the wells and incubated for three minutes at room temperature. At the end, 100 µL of stop solution was added to each well and mixed. The readings were obtained by using the Neogen^®^ Stat-Fax 4700 Microwell Reader, Lansing, MI, USA at 650 nm wavelength [23].

### 2.4. Detoxification of Aflatoxins

The contaminated samples were detoxified by the following methods.

#### 2.4.1. Physical Methods

In the physical methods, the detoxification was performed by washing the contaminated samples with cold and hot water. In the cold water treatment, 50 g of the samples with detectable aflatoxins was added to 500 mL conical flasks, 250 mL of water was added, and the flasks were shaken on a wrist-action shaker for 30 min. In the hot water treatment, 250 mL of water was added to 500 mL beakers containing 50 g of aflatoxin-contaminated samples and heated for 30 min at 100 °C [24].

#### 2.4.2. Chemical Methods

In the chemical methods, 50 g of the aflatoxin-contaminated samples was added to separate 500 mL conical flasks. The detoxification was performed by adding 10% (*w*/*v*) citric acid and 5% (*w*/*v*) sodium bicarbonate solutions to the aflatoxin-contaminated samples and shaking for 30 min in the case of 10% citric acid and incubating for 1 h for 5% sodium bicarbonate [25].

#### 2.4.3. Biological Methods

In the biological methods, 50 g of the aflatoxin-contaminated samples was added to separate 500 mL conical flasks. The detoxification was performed by adding 10% garlic paste and 10% kalonji oil to the aflatoxin-contaminated samples and incubating for 24 and 6 h for garlic and kalonji oil, respectively [26].

### 2.5. Quantification after Detoxification

The same ELISA technique was used to measure the concentration of aflatoxins in the samples that had been detoxified.

### 2.6. Statistical Analysis

Statistical analysis was conducted using IBM-SPSS software 29.0. A one-way ANOVA was used to determine the statistical significance between the treatments. The disparity in average values was assessed by Duncan’s multiple range test at a significance level of *p* < 0.05.

## 3. Results

### 3.1. Detection of Aflatoxins by Thin-Layer Chromatography

**Figure 1:**
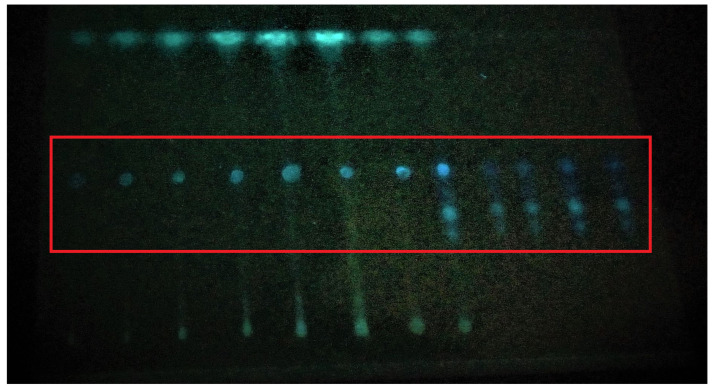
Detection of aflatoxins by thin-layer chromatography. The blue dots show the position of aflatoxins on TLC plate.

Aflatoxins were detected by thin-layer chromatography (Figure 1). Aflatoxins were detected in 35 samples out of a total of 56, while 21 wheat grain samples did not show any presence of aflatoxins (Table 1 and Table 2). The blue color on the TLC plate when seen under UV light indicated the presence of aflatoxins, which was further confirmed by spraying sulphuric acid.

### 3.2. Detection of Aflatoxins by Enzyme-Linked Immunosorbent Assay (ELISA)

**Figure 2:**
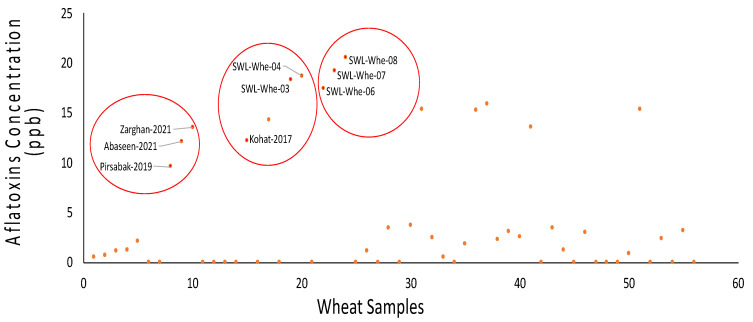
Detection of aflatoxins by ELISA. Scattergram showing the groups of wheat grain samples based on the concentration of aflatoxins.

Aflatoxins were detected in 35 out of 56 wheat grain samples, with concentrations ranging from 0.49 ppb to 20.56 ppb, while 21 samples did not show any presence of aflatoxins (Figure 2). The light and dark blue color of the wells indicated the presence of aflatoxins, which was further confirmed by the Neogen^®^ Stat-Fax 4700 Microwell Reader, Lansing, MI, USA.

### 3.3. Detoxification of Aflatoxins

The contaminated samples were detoxified by physical, chemical, and biological methods. The results of each of these methods are explained here.

#### 3.3.1. Physical Methods Revealed Reduction in Aflatoxin Concentrations

**Figure 3:**
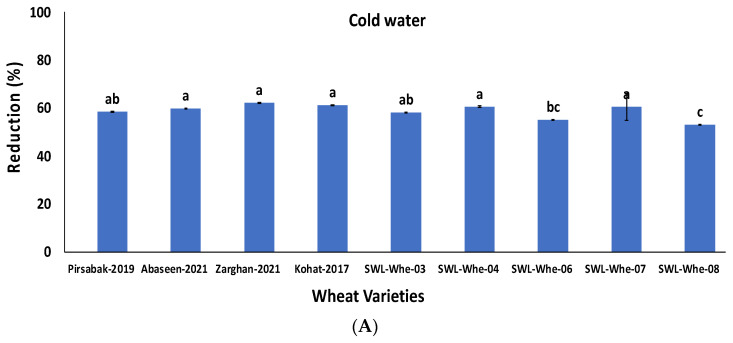

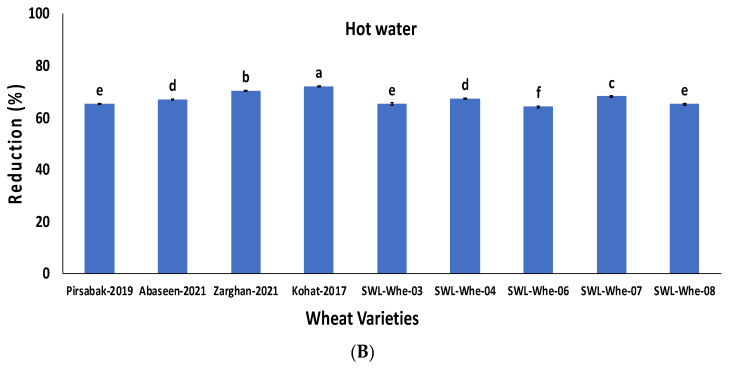
Percentage reduction in aflatoxin concentration after treatment with cold water (**A**) and hot water (**B**). The results are presented as the mean of three replicates, with vertical bars indicating ± standard deviation. Columns with identical letters do not show significant differences based on the LSD (least significant difference) test (*p* < 0.05).

Of the 35 wheat grain samples, the 9 highly contaminated wheat samples were washed with cold and hot water. All the samples showed significant reductions of 53–62% and 64–72% (*p* ≤ 0.05) in aflatoxins when treated with cold and hot water, respectively. The percentage reduction is shown in Figure 3A,B.

#### 3.3.2. Chemical Methods Greatly Detoxified the Aflatoxins

**Figure 4:**
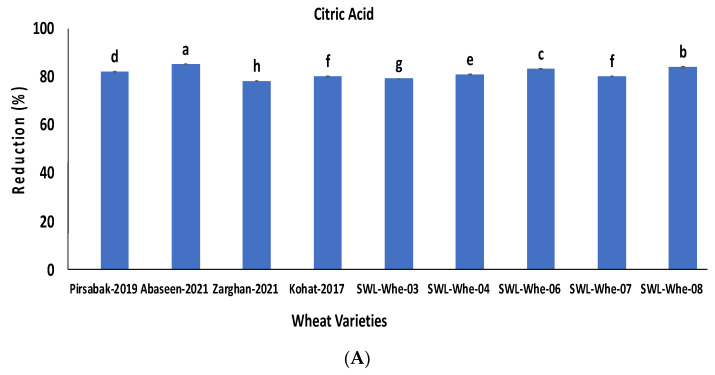

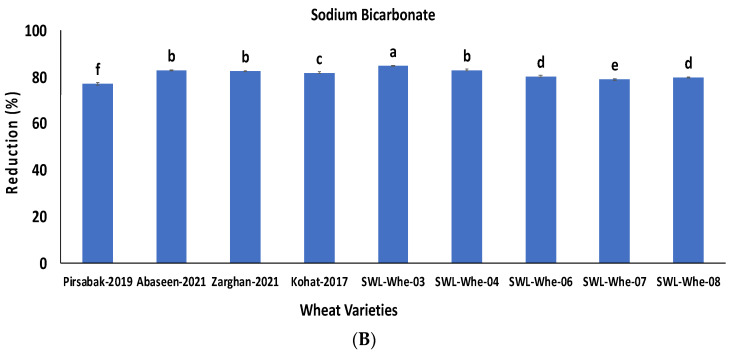
Percentage reduction in aflatoxin concentration after treatment with citric acid (**A**) and sodium bicarbonate (**B**). The results are presented as the mean of three replicates, with vertical bars indicating ± standard deviation. Columns with identical letters do not show significant differences based on the LSD test (*p* < 0.05).

The nine highly contaminated wheat samples were treated with 10% citric acid and 5% sodium bicarbonate solutions. All the wheat grain samples showed significant reductions in aflatoxins, ranging from 78 to 85% and 79 to 85% (*p* ≤ 0.05) with 10% citric acid and 5% sodium bicarbonate, respectively (Figure 4A,B).

#### 3.3.3. Biological Methods Were Found Effective for Reduction of Aflatoxins

**Figure 5:**
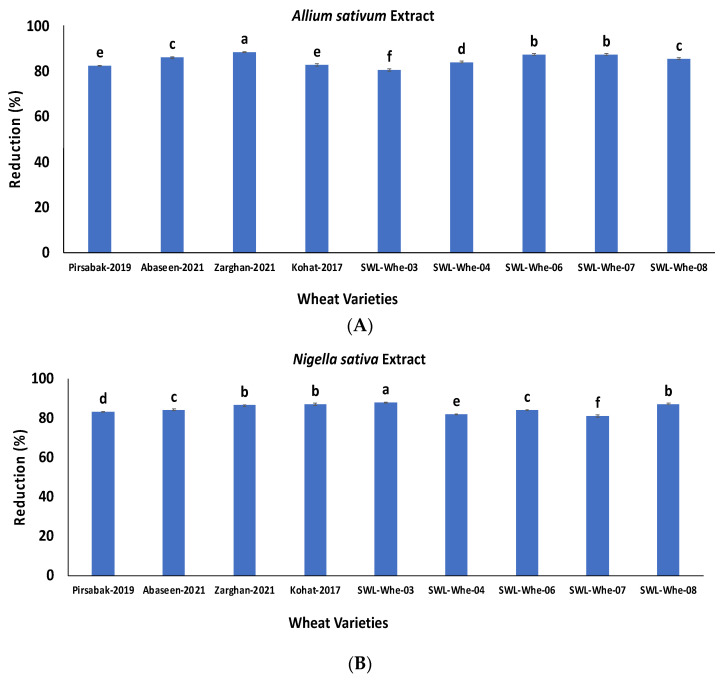
Percentage reduction in aflatoxin concentration after treatment with the extract of *Allium sativum* (**A**) and the extract of *Nigella sativa* (**B**). The results are presented as the mean of three replicates, with vertical bars indicating ± standard deviation. Columns with identical letters do not show significant differences based on the LSD test (*p* < 0.05).

The nine highly contaminated wheat samples were treated with garlic paste and kalonji oil. As a result, a significant reduction in aflatoxins ranging from 80 to 88% and 81 to 87% (*p* ≤ 0.05) was observed for garlic paste and kalonji oil, respectively (Figure 5A,B).

### 3.4. Comparison of Different Treatments’ Efficiency in Detoxification of Aflatoxins

**Figure 6:**
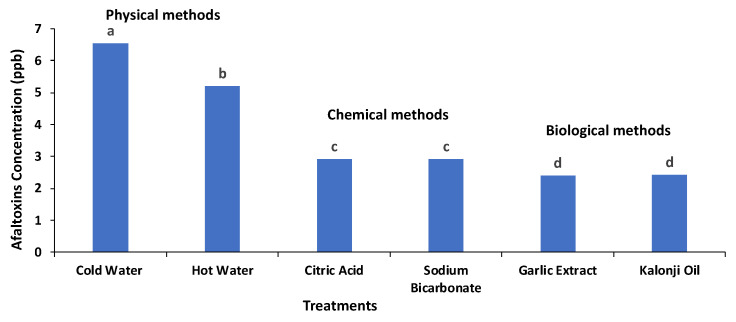
Aflatoxin detoxification efficiency of the tested treatments. Columns with identical letters do not show significant differences based on the LSD test (*p* < 0.05).

The efficiency of six different treatments in the detoxification of aflatoxins was compared. Although the physical treatments revealed a reduction in aflatoxin concentration, the reduction efficiency of the chemical and biological methods was found to be very effective. The treatment with *Allium sativum* (garlic) showed optimum results for the detoxification of aflatoxins as it greatly reduced the concentration of aflatoxins and did not change the organoleptic properties of the wheat. The aflatoxin detoxification efficiency of the six different treatments is shown in Figure 6.

### 3.5. Interactions of Different Treatments and Wheat Varieties

**Figure 7:**
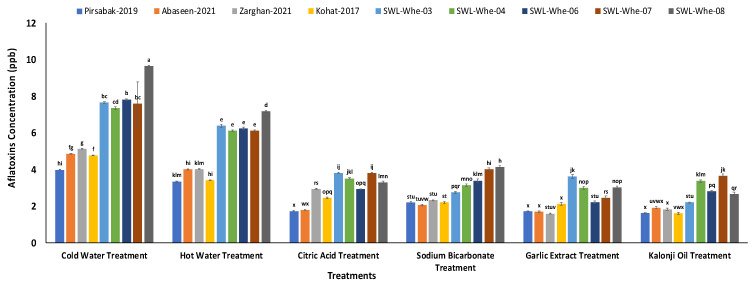
Interaction effects of different treatments and wheat varieties. Results are expressed as the mean of three replicates, and vertical bars represent ± standard deviation. Columns with identical letters do not show significant differences based on the LSD test (*p* < 0.05).

The efficiency of the different treatments against different wheat varieties was compared. All the treatments showed a significant reduction in the concentration of aflatoxins in different wheat varieties. The physical treatments showed aflatoxin reductions ranging from 53 to 72%, while the chemical and biological treatments showed reductions ranging from 78 to 85% and 80 to 88%, respectively. The interaction of the different treatments and varieties is shown in Figure 7.

## 4. Discussion

The fungal metabolites known as aflatoxins have the potential to be hazardous to human health. In this study, aflatoxins were identified in various wheat varieties from the Punjab and KPK provinces of Pakistan to assess the level of aflatoxin contamination [27]. After detection, the aflatoxins were detoxified by physical, chemical, and biological methods. Food commodities are being compromised globally by the contamination of different aflatoxins [28]. Huge losses of grains are experienced by Pakistan every year as a result of fungus infection. The agricultural sector aims to lower the annual losses of food crops. Due to the low production of valuable crops, both domestic and foreign trade is suffering significantly, which has reduced market value. The presence of aflatoxins in the environment has a negative impact on agricultural commodity availability, security, use, and stability, hence affecting food security [29]. A significant portion of the wheat crop is under stress due to fungal infestation. The legal limitations for aflatoxins in foods may vary from country to country depending on the economic conditions. The European Community’s Scientific Commission has enforced a 4.0 ppb maximum allowable limit for aflatoxins.

In this research, we detected aflatoxins in various wheat samples from Punjab and KPK provinces. For this purpose, 56 wheat grain samples were collected. These 56 samples were tested for the presence of aflatoxins by thin-layer chromatography and ELISA. Aflatoxins were detected in 35 samples (63%), ranging from 0.49 ppb to 20.56 ppb. The remaining 21 samples did not show any presence of aflatoxins. Out of the 35 contaminated samples, 20 samples (57%) were within the permissible limit according to the European Community’s Scientific Commission, and 15 samples (43%) were beyond the permissible level, which is in line with Hossain et al. [30] who detected aflatoxins in 42 out of 60 samples (70%). Zahra et al. [31] have reported that 20 out of 30 samples (66%) and 88 out of 100 samples (88%) were contaminated with aflatoxins. Out of the 35 contaminated wheat samples, 15 samples (43%) were above the permissible level for human consumption, which is in line with the results of Zahra et al. [31] who detected aflatoxin B1 in white and brown rice samples and stated that 67% of the brown rice and 56% of the white rice samples were contaminated with aflatoxin B1, and 43% of the contaminated brown rice and 23% of the contaminated white rice samples were above the permissible level for human consumption. Saima et al. [32] detected aflatoxin contamination in 124 wheat samples from North and South Punjab of Pakistan and stated that 71% of the wheat samples were contaminated with aflatoxins, ranging from 1.2 to 10 µg/kg. A study by Sabahat et al. [33] found that maize samples taken from various locations in Pakistan’s Faisalabad city had a mean AF concentration of 2 to 25 µg/kg. The presence of AFs in breakfast cereals and other items produced from wheat, such as pasta, spaghetti, macaroni salad, and lasagna, were demonstrated in two studies by Iqbal et al. [34]. In another study, it was found that out of all the products made from wheat, bucatini has the highest average level of aflatoxins, at 9.61 µg/kg. Aflatoxin contamination was also discovered in wheat bread samples from Spain, with concentrations ranging from 0.6 to 3.2 µg/kg [35]. Also, 26 samples of breakfast cereal from Portugal were found to have 0.003–0.130 µg/kg of aflatoxins [36]. Wheat from various parts of the world has different patterns of mycotoxin presence. Aflatoxin levels were observed to be higher in the current study compared to the studies conducted in some other countries. A study conducted by Serrano et al. [37] showed the incidence of NIV, FB2, and BEA to be 52.4%, 9.5%, and 14.3%, respectively, in samples of wheat grain from Spain, Italy, Morocco, and Tunisia. The contamination levels ranged from 339 to 679 ng of NIV, 121 to 158 ng of FB2, and 2.4 to 61.4 ng per kg of wheat. In addition, a study conducted by Andrade et al. [38] in Brazil has examined 55 samples of wheat products and confirmed DON contamination in all of them, with levels ranging from 79.7 to 916.1 g/kg. Additionally, the samples were contaminated with ZEA (84%), D3G (33%), FBs (7%), and OTA (4%), with contamination levels ranging from 17.8 to 205.6 g of ZEA, 54.8 to 335.2 g of D3G, 22.8 to 130 g of FBs, and 5.3 to 5.3 g of OTA per kg of sample. The different climates in different regions as well as genetic variability may be the cause of the variations in aflatoxin contamination in cereals.

The detoxification of aflatoxins was performed using physical, chemical, and biological methods. When aflatoxin-contaminated samples were washed with water, they showed a significant reduction in the concentration of aflatoxins, ranging from 53 to 62%, which is in line with Hwang and Lee [39] who detoxified aflatoxin-contaminated samples by 50–60%. Also, Saima et al. [32] detoxified aflatoxin contamination in wheat by 30 to 45% by washing with water. Jalili et al. [40] detoxified aflatoxins in black and white pepper by washing with water, with the reduction in the concentration of aflatoxins ranging from 14.7 to 15.3%. When we heated contaminated wheat samples in water at 100 ˚C for 30 min, it showed a significant reduction in the concentration of aflatoxins, ranging from 64 to 72%, which is in line with the study conducted by Saima et al. [32] who detoxified aflatoxins in wheat (30–45%), rice (45–51%), and maize (30–40%) by heating.

In this research, aflatoxin-contaminated samples were treated with citric acid and sodium bicarbonate. Both treatments showed significant reductions in aflatoxin concentration, ranging from 77 to 85%, which is in line with a previous study by Nazir et al. [41] and Iqbal et al. [34] who showed significant reductions in aflatoxins ranging from 65 to 80% and 70 to 82%, respectively. Citric acid has a very diverse and complicated chemical makeup, but the main components are monoterpenes and oxygenated compounds. Limonene was found to be a more effective antifungal by Negro et al. [42]. According to Martos et al. [43], phenolic chemicals interact with the polar region of the membrane, causing the membrane to degrade and the fungal cells to die as a result. Safara et al. [44] detoxified aflatoxins by 90–97.7% by using 1N aqueous citric acid solution. Abuagela et al. [45] detoxified aflatoxins in peanuts by 97-98% using citric acid combined with pulsed light treatment. Summia et al. [46] detoxified aflatoxins in poultry feed by 44–54.3% using 10% citric acid solution, which is in line with our study. Castro-Rios et al. [47] achieved detoxification of aflatoxins (70–100%) in corn by using sodium bicarbonate, which is in line with the current study.

This study demonstrates the antifungal activity of *Allium sativum* extracts against *A. flavus*, and during detoxification, a significant reduction in aflatoxins ranging from 81 to 88% was observed. Various authors have reported the effectiveness of *Allium sativum* vegetable extracts against mold species. At a concentration of 5 ppb, *A. sativum* specifically reduced the growth of *A. flavus* (65–78%) and the production of aflatoxins from 12.2 to 15.7%. Organosulfur components in garlic, like diallyl sulphide and ajoene, prevent DNA from binding to aflatoxin. *Allium sativum* contains a variety of antioxidant properties, and it has been used to treat hyperlipidemia, thrombus formation, and irregular heartbeat. Roheela et al. [48] concluded that garlic is the best antifungal agent that has the capability to reduce the growth of *Aspergillus niger* and *Aspergillus flavus*. Ziaur et al. [49] concluded that garlic exhibits a high potential against various types of bacterial and fungal pathogens. Gaber et al. [50] studied the chemical constituents and pharmacological activities of garlic (*Allium sativum* L.) and stated that *A. sativum* has various biological activities including antibacterial, antiviral, antifungal, antiprotozoal, antioxidant, anti-inflammatory, and anticancer activities. The plants that we used in this research (*Allium sativum* and *Nigella sativa*) have already been characterized by various authors, and they have evaluated the antifungal activities of these plants against Aspergillus species, which is why we have not characterized these plants again, and we believe that the detoxification of aflatoxins was due to the antifungal activity of these plants. The black seed oil contains a variety of bioactive substances as well as several antioxidants with strong inhibitory properties. Numerous past studies have shown that essential oils have the ability to completely eradicate fungus, for example, *Curcuma longa* L. [51], *Santolina chamaecyparissus* [52], *Mentha cardiac* L. [53], *Melissa officinalis* [54], *Nectandra* [55], *Cinnamomum glaucescens* [56], *Mikania scanders* [57], and *Michelia alba* [58]. Some studies have also reported how well the antifungal properties worked against a certain type of fungus such as wood-rot fungi, *Aspergillus flavus* [59], and *Aspergillus niger* [60]. Black seed oil was very effective and decreased aflatoxin contamination by up to 87%, which is similar to the results produced by Nazir et al. [61] in which the aflatoxins were reduced by up to 100%. Black seed oil’s antifungal properties show considerable potential for *A. flavus* prevention. Black seed oil’s MIC value is significantly lower than that of other essential oils, making it economically viable for the suppression of food-borne fungi [62]. Kedia et al. [63] found that 0.6 µL/ml of black seed oil inhibited 100% of several *Aspergillus* species. Studies have revealed that black seed oil lowers the amount of ergosterol, a fundamental element of the fungal cell membrane that also contributes to the preservation of cell integrity and functionality. The amount of fungal biomass decreases as a result of the total suppression of ergosterol production [63]. Another study by Martos et al. [43] found that 0.94% of essential oil content can completely inhibit the growth of *Aspergillus niger*, *Aspergillus flavus*, *Penicillium verrucosum*, and *P. chrysogenum.* All the treatments (physical, chemical, biological) reduced the concentration of aflatoxins; however, the biological treatment was the best as it greatly reduced the concentration of aflatoxins and did not affect the organoleptic properties of the wheat.

## 5. Conclusions

The difference in aflatoxin concentration in different wheat varieties could be due to genetic variations. Among the different methods for the detoxification of aflatoxins in wheat, the biological method could be the method of choice as it greatly reduced the aflatoxin concentration with no harmful effects on the quality of the grains. The current study revealed these findings, which have been reported before for Pakistani wheat varieties under diverse environmental conditions. To avoid aflatoxin contamination, it is required to understand the molecular and genetic basis of aflatoxin production, improve management strategies, better allocate monitoring efforts, and modify current agronomic practices. While genetic variation may indeed play a role in aflatoxin susceptibility among wheat varieties, the scope of the current study was limited to evaluating the treatment efficacy of aflatoxin-contaminated samples. Future research could explore the genetic underpinnings of aflatoxin susceptibility in wheat varieties in more detail.

## Figures and Tables

**Table 1:** Detection of aflatoxins by TLC in selected wheat varieties of District Kohat, KPK.

Wheat Varieties	Aflatoxins	Wheat Varieties	Aflatoxins
Shahkar-2013	+	Abaseen-2021	+
Zincol-2016	+	Zarghan-2021	+
Pirsabak-2021	+	Dilkash-20	-
Khaista-2017	+	Pirsabak-2015	-
Markaz-2019	+	Gulzar-2019	-
Fahim-2019	-	Subhani-2021	-
Paseena-2017	-	Kohat-2017	+
Pirsabak-2019	+	Taskeen-2021	-

**Table 2:** Detection of aflatoxins by TLC in wheat samples of District Sahiwal, Punjab.

Wheat Samples	Aflatoxins	Wheat Samples	Aflatoxins
SWL-Whe-01	+	SWL-Whe-21	+
SWL-Whe-02	*	SWL-Whe-22	+
SWL-Whe-03	+	SWL-Whe-23	+
SWL-Whe-04	+	SWL-Whe-24	+
SWL-Whe-05	-	SWL-Whe-25	+
SWL-Whe-06	+	SWL-Whe-26	-
SWL-Whe-07	+	SWL-Whe-27	+
SWL-Whe-08	+	SWL-Whe-28	+
SWL-Whe-09	-	SWL-Whe-29	-
SWL-Whe-10	+	SWL-Whe-30	+
SWL-Whe-11	-	SWL-Whe-31	-
SWL-Whe-12	+	SWL-Whe-32	-
SWL-Whe-13	-	SWL-Whe-33	-
SWL-Whe-14	+	SWL-Whe-34	+
SWL-Whe-15	+	SWL-Whe-35	+
SWL-Whe-16	+	SWL-Whe-36	-
SWL-Whe-17	+	SWL-Whe-37	+
SWL-Whe-18	-	SWL-Whe-38	-
SWL-Whe-19	+	SWL-Whe-39	+
SWL-Whe-20	+	SWL-Whe-40	-

## Data Availability

Data will be made available on request.
